# Revealing the Intrinsic Peroxidase-Like Catalytic Mechanism of Heterogeneous Single-Atom Co–MoS_2_

**DOI:** 10.1007/s40820-019-0324-7

**Published:** 2019-11-22

**Authors:** Ying Wang, Kun Qi, Shansheng Yu, Guangri Jia, Zhiliang Cheng, Lirong Zheng, Qiong Wu, Qiaoliang Bao, Qingqing Wang, Jingxiang Zhao, Xiaoqiang Cui, Weitao Zheng

**Affiliations:** 1 Key Laboratory of Automobile Materials of MOE, School of Materials Science and Engineering, Jilin University, 2699 Qianjin Street, Changchun, 130012 People’s Republic of China; 2 Department of Materials Science and Engineering, ARC Centre of Excellence in Future Low-Energy Electronics Technologies (FLEET), Monash University, Clayton, VIC 3800 Australia; 3 Key Laboratory of Photonic and Electronic Bandgap Materials, Ministry of Education, College of Chemistry and Chemical Engineering, Harbin Normal University, Harbin, 150025 People’s Republic of China; 4 Department of Bioengineering, University of Pennsylvania, 210 South 33rd Street, 240 Skirkanich Hall, Philadelphia, PA 19104 USA; 5 Beijing Synchrotron Radiation Facility, Institute of High Energy Physics, Chinese Academy of Sciences, Beijing, 100190 People’s Republic of China; 6 School of Chemistry and Chemical Engineering, MOE Key Laboratory of Micro-System and Micro-Structure Manufacturing, Harbin Institute of Technology, Harbin, 150001 People’s Republic of China

**Keywords:** Biocatalysis, Nanozymes, Peroxidase mimic, Reaction mechanisms, Single-atom catalysts

## Abstract

The single-atom nanozyme is a new concept and has tremendous prospects to become a next-generation nanozyme. However, few studies have been carried out to elucidate the intrinsic mechanisms for both the single atoms and the supports in single-atom nanozymes. Herein, the heterogeneous single-atom Co–MoS_2_ (SA Co–MoS_2_) is demonstrated to have excellent potential as a high-performance peroxidase mimic. Because of the well-defined structure of SA Co–MoS_2_, its peroxidase-like mechanism is extensively interpreted through experimental and theoretical studies. Due to the different adsorption energies of substrates on different parts of SA Co–MoS_2_ in the peroxidase-like reaction, SA Co favors electron transfer mechanisms, while MoS_2_ relies on Fenton-like reactions. The different catalytic pathways provide an intrinsic understanding of the remarkable performance of SA Co–MoS_2_. The present study not only develops a new kind of single-atom catalyst (SAC) as an elegant platform for understanding the enzyme-like activities of heterogeneous nanomaterials but also facilitates the novel application of SACs in biocatalysis.

**Electronic supplementary material:**

The online version of this article (10.1007/s40820-019-0324-7) contains supplementary material, which is available to authorized users.

## Highlights

- Single-atom Co–MoS_2_ (SA Co–MoS_2_) is prepared successfully to serve as a proof-of-concept nanozyme model, which exhibits peroxidase-like performance comparable to that of natural enzymes.
- The different mechanisms between the single-atom metal center and the support are investigated experimentally and theoretically.

**Electronic supplementary material:**

The online version of this article (10.1007/s40820-019-0324-7) contains supplementary material, which is available to authorized users.

## Graphical abstract

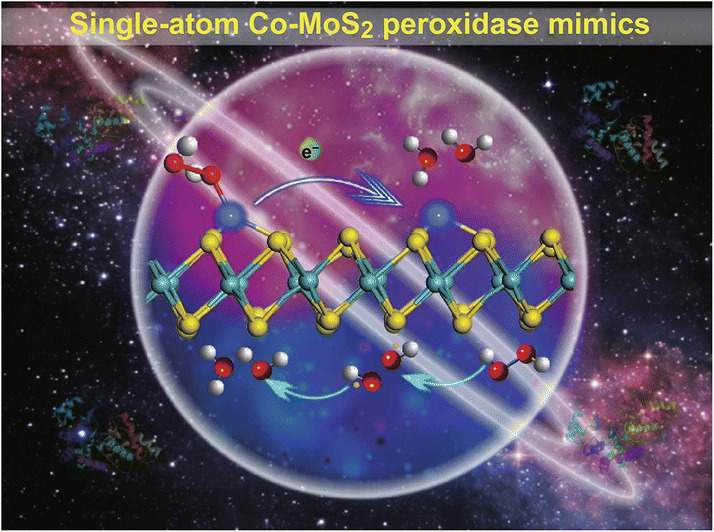

## Introduction

The high extraction cost and low stability of natural enzymes caused by denaturation and deactivation hinder their practical applications [1]. Nanomaterials with enzyme-like characteristics (nanozymes) have become promising alternatives to traditional enzymes and have the unique advantage of bridging nanotechnology and biology [2, 3]. Many types of nanozymes have been reported and applied in the catalysis of a broad range of reactions [4–6]. However, the activity and specificity of nanozymes still differ substantially from those of natural enzymes. There is an urgent need to obtain a deep understanding of the intrinsic catalytic mechanisms and kinetics to design and develop more efficient enzyme mimic systems, but meeting this need remains a significant challenge [7].

The atomistic-level exploration of the original active sites of nanozymes is mainly performed by simplifying the single-component catalysts of noble metal nanoparticles [8–10], transition metal oxides [11], and carbon-based materials [12, 13]. The cascade reactions catalyzed by multiple enzymes in an organism provide great impetus for preparing composite catalysts by integrating different kinds of nanozymes to mimic the complexity of biocatalysis [14, 15]. Moreover, natural horse radish peroxidase is composed of a heme group and two calcium atoms, and the integrity of this complex protein is crucial for the high selectivity and efficiency of the natural homogeneous enzyme [16]. However, the structure of heterogeneous nanozymes is too inhomogeneous to achieve satisfactory enzyme-like activity and selectivity. In addition, the internal relationship between homogeneous molecular enzymes and heterogeneous nanozymes is still poorly understood and poses a challenge for achieving an in-depth understanding of enzyme-like catalytic mechanisms. Single-atom catalysts (SACs), which act as a bridge between homogeneous and heterogeneous catalysis, not only offer a simple ideal model for the design of rational nanozymes at the atomic level but also provide an appropriate platform for studying the origins of enzyme-like activities thanks to their advantages of homogeneous active sites and single atoms in low-coordination environments [17–19]. However, studies on single-atom nanozymes are confined to only single-atomically dispersed metal-N_*x*_ on a carbon support (M–N-C) [20–25]. Further systematic studies are required to develop more kinds of heterogeneous single-atom catalysts and deeply investigate the enzyme-like catalytic mechanisms for both the single atoms and the supports.

Herein, we chose single-atom Co–MoS_2_ (SA Co–MoS_2_) as a proof-of-concept nanozyme for examination of a high-performance peroxidase-like reaction. Through experiments and calculations, we explored in detail how the introduction of SA Co affected the peroxidase-like activity, substrate affinity, and corresponding reaction energies. The results of these studies facilitated the identification of the different catalytic sites in the hybrid SA Co–MoS_2_ catalyst and the reaction mechanism. This research may enhance the knowledge on the intrinsic properties of enzyme-like activities and pave the way for the rational design of highly effective nanozymes.

## Experimental Section

### Chemicals and Materials

All chemicals were used as received without further purification. H_2_O_2_ (30 wt%), 3,3′,5,5′-tetramethylbenzidine (TMB), *o*-phenylenediamine (OPD), 2,2′-azinobis(3-ethylbenzthiazoline-6-sulfonate) (ABTS), horse radish peroxidase (HRP), phosphate-buffered saline (PBS) (0.1 M), *D*-(+)-glucose, terephthalic acid (TA), uric acid (UA), *L*-ascorbic acid (AA), dopamine hydrochloride (DA), cytochrome *c* (Cyt *c*, ≥ 95%), trioctylphosphine oxide (TOPO, C_24_H_51_OP, 90%), anhydrous *o*-dichloride benzene (DCB, C_14_H_10_Cl_2_N_2_), and cobalt carbonyl (Co_2_(CO)_8_, Co content ≥ 90%) were obtained from Sigma-Aldrich (USA). Nafion-ethanol solution was purchased from Adamas-beta Chemical Co., (Switzerland). Ammonium heptamolybdate tetrahydrate ((NH_4_)_6_Mo_7_O_24_·4H_2_O, AR), thiourea (CH_4_N_2_S, AR), *n*-hexane (C_6_H_14_, AR), potassium thiocyanate (KSCN, AR), sodium acetate (NaAc, AR), sodium chloride (NaCl), fructose, lactose, xylose, l-cysteine, mannose, sucrose, urea, serine, arginine, cysteine, acetic acid (HAc, AR), ethanol (CH_3_CH_2_OH, AR), and dimethyl sulfoxide (DMSO) were purchased from Sinopharm Chemical Reagent (China). Oleylamine (OA, C_18_H_37_N, 80–90%) was purchased from Acros Organics, USA. Argon (Ar, ultra-purity) was obtained from Xin’guang Gas Co., China. Carbon fiber paper was purchased from AvCarb, USA. Deionized water from a Milli-Q system (18.2 MΩ cm at 25 °C) was used throughout the experiments.

### Characterization

Transmission electron microscopy (TEM), high-resolution transmission electron microscopy (HRTEM), and energy-dispersive X-ray (EDX) spectroscopy were performed on an FEI Tecnai G2 F20 transmission electron microscope operated at 200 kV. The samples were prepared by dropping ethanol dispersions of the samples onto 300-mesh carbon-coated copper grids and then evaporating the solvent. HRTEM images were acquired using a JEOL-ARM200F transmission electron microscope operated at 200 kV. The attainable spatial resolution of the microscope was 78 pm with a probe spherical aberration corrector. High-angle annular dark field (HAADF) images were acquired with an illumination semi-angle of 25 mrad and probe current of 100 pA. The dwell time for image acquisition was set at 10 μs per pixel to ensure the desirable signal-to-noise ratio. The collection angles for the HAADF images were fixed at 90–250 mrad. To obtain high-quality STEM images with atomic resolution, SA Co–MoS_2_ was pre-treated at 80 °C in a vacuum oven for 4 h to remove any organic ligands from its surface. X-ray photoelectron spectroscopy (XPS) measurements were performed with an ESCALAB-250 instrument (Thermo Fisher Scientific, USA) using a monochromatic Al-Kα (14, 866 eV) radiation source and a hemisphere detector with an energy resolution of 0.1 eV. Peak positions were corrected by the C 1 s spectrum at 284.6 eV. Co K-edge X-ray absorption spectra were obtained at Beijing Synchrotron Facility (BSRF) on beamline 1 W1B (XAFS station) at 2.2 GeV in fluorescence mode. The EXAFS raw data were processed using the ATHENA module implemented in the IFEFFIT software packages according to the standard procedures of the background-subtracted, normalized and Fourier transformed. The Raman spectra were obtained by a Renishaw 1000 spectrometer with an excitation laser line of 532 nm. Typical UV–Vis spectra were acquired with a UV-2550 spectrometer (Shimazu Co., Japan). Fluorescence (PL) spectra were obtained using an LS55 spectrometer (PerkinElmer Inc., USA). Electrochemical tests were carried out on a CHI 650D electrochemical workstation (Shanghai, Chenhua Co., China). Optical photographs were acquired using an iPhone 6s.

MoS_2_ was synthesized according to the reported procedure [26]. 24.7 mg of hexaammonium heptamolybdate tetrahydrate and 53.2 mg of thiourea were dissolved in 20 mL of deionized water under vigorous stirring. Then, the solution was transferred into a 30 mL Teflon-lined stainless steel autoclave and maintained at 220 °C for 18 h before the reaction system was allowed to cool down to room temperature. The final product was washed several times with water and ethanol to remove any possible ions and finally suspended in ethanol.

Co nanodisks (NDs) were synthesized using standard air-free procedures [27]. 0.1 g of TOPO was degassed with argon for 20 min in a three-neck flask, following by the introduction of 0.1 mL of OA and 15 mL of DCB under argon, then the regents were heating to reflux (182 °C) and rapidly injecting 0.54 g of Co_2_(CO)_8_ containing 1–5% hexane diluted in 3 mL of DCB (precursor solution). 10 min later, the reaction was stopped by quenching in an ice water bath. The final products were processed by extracting the solution, washing several times with water and methanol to remove the possible ions and organic component, and finally suspending in argon-saturated water for storage.

### Preparation of SA Co–MoS_2_ Catalysts

Assembly of Co NDs onto MoS_2_ [28]. Co NDs were assembled on MoS_2_ nanosheets by mixing the two solutions in a 20 mL conical flask with certain mass ratio and following by 20 min degassing with argon. The mixture solution was ultrasonicated for 24 h (800 W at 40 KHz) while keeping at 4 °C in a thermostat system. The product was centrifuged and washed for three times with water, and finally stored as argon-saturated water suspension.

The working electrode was prepared by drop casting Co ND/MoS_2_ onto the carbon fibre paper. The electrochemical leaching was performed by cyclic voltammetry scanning between 0.1 V to − 0.4 V in a 0.5 M H_2_SO_4_ solution for 50 cycles. The final SA Co-MoS_2_ was obtained by sonicating the electrode in an ethanol solution for 30 min and then collecting the sedimentation after 6 h. The whole leaching process was repeated twice to ensure the entirely removing of Co nanodisks.

### Peroxidase-Like Activity of SA Co–MoS_2_

The working stock solution of 5.0 M H_2_O_2_ was prepared by diluting H_2_O_2_ (30 wt%), and solutions of different concentrations were prepared by sequential dilution of the stock solution. The peroxidase-like activity of SA Co–MoS_2_ was examined in the oxidation of TMB, ABTS, and OPD, which produced colorimetric changes with different typical UV–Vis absorbance peaks. The reactions were carried out as follows: 8 μL of SA Co–MoS_2_ catalyst (1.25 mg mL^−1^), 16 μL of TMB (10 mM), and 326 μL of acetate buffer (0.20 mM, pH = 4.0) were mixed. Then, 50 μL of a H_2_O_2_ solution of different concentrations was added to keep the total volume at 400 μL. Finally, the mixture was further incubated at 45 °C for 20 min, and a blue color with major absorbance peaks at 370 and 652 nm was produced.

### OH Radical and Dissolved O_2_ Measurement

The fluorescence spectra were obtained under the following conditions: 50 mM H_2_O_2_, 1 mM terephthalic acid (TA), and different concentrations of the SA Co–MoS_2_ catalyst were first incubated in 0.1 M HAc-NaAc buffer (pH = 5.0) at 40 °C for 8 h, and then the catalysts were then removed from the reaction solution by centrifugation. The final solutions were used for fluorometric measurements.

### Apparent Kinetic Analysis

Steady-state kinetic assays of the TMB/H_2_O_2_/SA Co–MoS_2_ reaction system were performed by recording the absorption spectrum at selected time intervals in scanning kinetic mode in the above optimized conditions [29]. The TMB concentration was determined from its oxidation products and absorbance values monitored at 652 nm, which were converted by the Beer–Lambert Law as follows: *A* = *εbc*, where the molar absorption coefficient *ε* was 39,000 M^−1^ cm^−1^ and the path length *b* of the glass cuvette was 0.1 cm. For TMB and H_2_O_2_ as substrates, the apparent kinetic parameters were fitted based on the double-reciprocal plot derived from the Michaelis–Menten equation as follows: $1/\nu \, = \,\left( {K_{\text{m}} /V_{\hbox{max} } } \right)\, \times \,\left( {1/[S]} \right)\, + \,1/V_{\hbox{max} }$, where *ν* is the reaction initial velocity, *V*_max_ is the maximal reaction velocity, [*S*] is the concentration of the substrate, and *K*_m_ is the Michaelis–Menten constant.

### Preparation of SA Co–MoS_2_/GCE

Before modification of a glassy carbon electrode (GCE, *d* = 3 mm), the bare GCE was carefully polished with emery paper and chamois leather containing 1.0, 0.3, and 0.05 μm alumina slurries to obtain a mirror-like surface and then sequentially rinsed with acetone, ethanol, and ultrapure water in an ultrasonic bath. After polishing, the electrode was immersed in 0.50 M H_2_SO_4_ for activation by 10 cycles between − 0.20 and 0.60 V with a scan rate of 50 mV s^−1^ until a steady profile was obtained. After the electrode was dried under a nitrogen flow, 5 μL of a dispersion of SA Co–MoS_2_ or MoS_2_ was coated on the surface of the electrode and dried under ambient conditions. The doses of the SA Co–MoS_2_ and MoS_2_ catalysts were kept constant according to the amount of Mo (17.6 μg cm^−2^ for Mo) determined by inductively coupled plasma mass spectrometry (ICP-MS). Then, 6 μL of a Nafion − ethanol solution (0.05 wt%) was pipetted onto the electrode surface.

### Electrochemical Measurements

PBS buffer (pH = 7.4) was bubbled with high-purity nitrogen for at least 10 min to deoxygenate it prior to each test. Electrochemical measurements were carried on a computer-controlled CHI650D electrochemical workstation with a standard three-electrode system at room temperature. A bare GCE or various modified GCEs served as the working electrode (WE). A platinum foil electrode (1 cm × 1 cm) was used as the counter electrode, and a saturated calomel electrode (SCE, Hg/HgCl_2_ in saturated KCl) was used as the reference electrode. Before adding H_2_O_2_, all modified GCEs were cleaned by cyclic voltammetric (CV) sweeping from − 0.7 to 0.7 V at 50 mV s^−1^ in electrolyte for 40 cycles.

### DFT Calculations

Our spin-polarized DFT computations were performed by using the DMol^3^ code [30, 31]. The electron interactions were described by Perdew–Burke–Ernzerhof (PBE) exchange–correlation functional within the generalized gradient approximation (GGA) [32] The empirical correction in Grimme’s scheme (i.e., DFT + D2) was utilized to treat the (possible) van der Waals interactions [33] The relativistic effects of transition metals were considered through the density functional semi-core pseudopotential (DSPP) [34], in which a single effective potential and some degree of relativistic corrections replace the core electrons, while the double numerical plus polarization (DNP) basis set was used for other elements. Self-consistent field (SCF) computations were performed with a convergence criterion of 10^−6^ a.u. on the total energy and electronic computations. To ensure high-quality results, the real-space global orbital cutoff radius was chosen as high as 5.2 Å in all the computations. The Brilliouin zone was sampled with a Monkhorst–Pack mesh with a 5 × 5 × 1 grid in reciprocal space during geometry optimizations. The 1T- and 2H-MoS_2_ nanosheets were built using a 4 × 4 supercell containing 16 Mo and 32 S atoms. The vacuum space was set to 20 Å, which was enough to avoid the interactions between periodic images.

The Gibbs free energy change (Δ*G*) of every elemental step was determined by the following equation: Δ*G* = Δ*E* + Δ*ZPE* – *T*Δ*S*. The reaction energy (Δ*E*) can be directly determined by analyzing the DFT total energies. For example, the adsorption energy of H_2_O_2_ (*E*_ad_) on substrate was defined as: $E_{\text{ad}} = E_{{{\text{substrate-H}}_{2} {\text{O}}_{2} }} - E_{\text{substrate}} - E_{{{\text{H}}_{2} {\text{O}}_{2} }}$ , where $E_{{{\text{substrate-H}}_{2} {\text{O}}_{2} }}$, *E*_substrate_, and $E_{{{\text{H}}_{2} {\text{O}}_{2} }}$ are the DFT total energies for the H_2_O_2_ adsorbed substrate, free substrate, and H_2_O_2_ molecule, respectively. ∆*E*_ZPE_ and ∆*S* are the zero point energy difference and the entropy difference between the adsorbed state and the gas phase, respectively, and *T* is the system temperature (298.15 K, in our work). For each system, its *E*_zpe_ can be calculated by summing vibrational frequencies over all normal modes *ν* (*E*_zpe_ = 1/2Σ*ħν*). The entropies of the free molecules (H_2_O_2_, H_2_, and H_2_O) were taken from the NIST database, while the energy contribution from the adsorbed state is neglected. One-half of the chemical potential of H_2_ molecule was used as the chemical potential of the proton–electron pairs. In addition, the conductor-like screening model (COSMO) was used to simulate the H_2_O solvent conditions, and its dielectric constant was 78.54.

## Results and Discussion

### Characterization of SA Co–MoS_2_

**Fig. 1:**
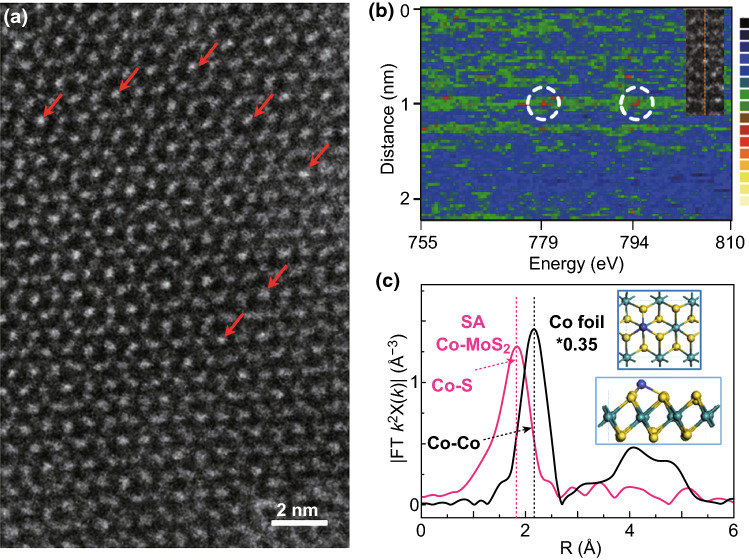
**a** Aberration-corrected HAADF-STEM image of SA Co–MoS_2_. **b** EELS results of SA Co–MoS_2_. **c** FT-EXAFS spectra of SA Co–MoS_2_ and cobalt foil at the Co K-edge. The inset shows the atomic structure of SA Co–MoS_2_. Yellow spheres: S; aqua spheres: Mo; slate blue spheres: Co. (Color figure online)

SA Co–MoS_2_ was obtained via our recently developed assembly leaching method. The as-synthesized material had the form of ultrathin nanosheets with no Co nanoparticles or clusters on the surface (Fig. S1a), which was also further validated by XRD patterns (Fig. S1b). From the images of EDX, Co was uniformly distributed over the whole catalyst (Fig. S1c). More interestingly, the high-resolution TEM images showed that the introduction of Co atoms induced the crystal lattice of MoS_2_ from the well-organized to disordered arrangement (Fig. S2). Highly abundant single Co atoms were clearly observed by high-angle annular dark field scanning transmission electron microscopy (HAADF-STEM), as indicated by the bright spots marked by red arrows in Fig. 1a. The line profile from atomically sensitive electron energy loss spectroscopy (EELS) provided Co L_2_ and L_3_ edges of 794 and 779 eV, respectively (Fig. 1b) [35]. The XPS results of Co 2p confirmed that the major state of SA Co–MoS_2_ was Co^3+^ (Fig. S3). The Fourier transform of the extended X-ray absorption fine structure (FT-EXAFS) analysis of SA Co–MoS_2_ exhibited a strong peak at 1.79 Å assigned to Co–S coordination (Fig. 1c and S4a) without Co–Co bond contributions [36]. The formation of a Co–S bond and lattice mismatch induced the phase change of MoS_2_ from the 2H phase to the distorted 1T phase, which was verified by the appearance of new vibration characteristic peaks of 1T-MoS_2_ in Raman spectra (Fig. S4b) [37]. Based on the quantitative EXAFS fitting analysis in *k* and *R* spaces (Fig. S5 and Table S1), the average Co–S coordination number within the SA Co–MoS_2_ is 3.4 ± 0.6 [38]. These results demonstrated the successful construction of a model SA Co–MoS_2_ catalyst in which a single Co atom sat on top of the Mo atoms in 1T, and MoS_2_ was coordinated by three adjacent sulfur atoms (the inset of Fig. 1c) [39].

### The peroxidase-like Activity of SA Co–MoS_2_

**Fig. 2:**
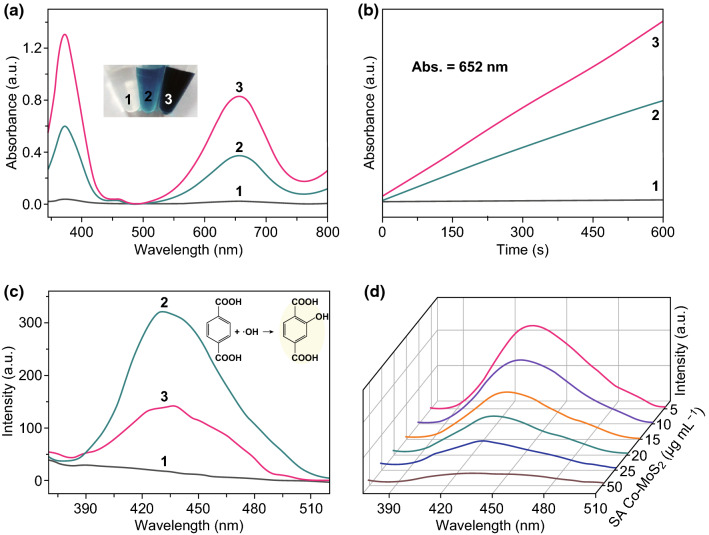
**a** UV–Vis spectra of different reaction systems: (1) TMB + H_2_O_2_, (2) TMB + H_2_O_2_ + MoS_2_, and (3) TMB + H_2_O_2_ + SA Co–MoS_2_. **b** The time-dependent absorbance changes at 652 nm and an optical image depicting the color changes. **c** The formation of ·OH using TA as a fluorescent probe in different reaction systems: (1) TA + H_2_O_2_, (2) TA + H_2_O_2_ + MoS_2_, and (3) TA + H_2_O_2_ + SA Co–MoS_2_. **d** Effects of the concentration of SA Co–MoS_2_ on the changes in ·OH with TA as the fluorescence probe. (Color figure online)

The catalysis of the 3,3′,5,5′-tetramethylbenzidine (TMB)-H_2_O_2_ reaction system was assayed and optimized (Fig. S6) to evaluate the peroxidase-mimicking ability of SA Co–MoS_2_ [29, 40]. The addition of SA Co–MoS_2_ resulted in a much stronger absorbance at 652 nm and yielded a more obvious blue color than the addition of pristine MoS_2_, as shown in Fig. 2a, b. The enhanced peroxidase-like activity of SA Co–MoS_2_ was further confirmed by the electrochemical response to the oxidation of TMB by H_2_O_2_ (Fig. S7) and proven with other chromogenic peroxidase substrates, *o*-phenylenediamine (OPD) (Fig. S8a) and 2,2′-azinobis(3-ethylbenzthiazoline-6-sulfonate) (ABTS) (Fig. S8b) [39]. The catalytic activity of SA Co–MoS_2_ was dependent on the pH, temperature, and H_2_O_2_ concentration but was less sensitive to these factors than was horseradish peroxidase (HRP) (Fig. S9). The typical Michaelis–Menten curves for H_2_O_2_ (Fig. S10a) and TMB (Fig. S10b) were measured and then fitted to Lineweaver–Burk plots, from which the apparent steady-state kinetic parameters were determined and compared with those of HRP (Table S2) [41]. The double-reciprocal plots obtained by varying the H_2_O_2_ concentration with a fixed concentration of TMB (Fig. S10c) or vice versa (Fig. S10d) showed nearly equal slopes, which was characteristic of a typical ping-pong mechanism, as was observed for HRP. All the above experimental results demonstrated that SA Co–MoS_2_ could serve as a peroxidase mimic with high activity and stability. According to previous reports, the peroxidase mimic-mediated activity of MoS_2_ depended on the amount of hydroxyl radical (·OH) produced by the Fenton-like mechanism [42]. Therefore, terephthalic acid (TA) was used as a fluorescent probe to detect ·OH during the reaction, where TA easily captured ·OH and generated highly fluorescent 2-hydroxy terephthalic acid, which exhibited emission at approximately 425 nm [43] As shown in Fig. 2c, the fluorescence at 425 nm was remarkably enhanced by both MoS_2_ and SA Co–MoS_2_, indicating that more ·OH was produced in the presence of the catalysts. However, the MoS_2_ system showed much stronger fluorescence than the SA Co–MoS_2_ system, which was opposite the trend in the corresponding peroxidase-like performance shown in Fig. 2c. This phenomenon suggested that the increased catalytic activity of SA Co–MoS_2_ toward H_2_O_2_ did not rely on the Fenton-like reaction [44, 45]. The fluorescence intensity gradually decreased as the concentration of SA Co–MoS_2_ continued to increase (Fig. 2d), which further indicated that the introduction of SA Co exhibited ·OH scavenging properties rather than generating ·OH [46–48].

**Fig. 3:**
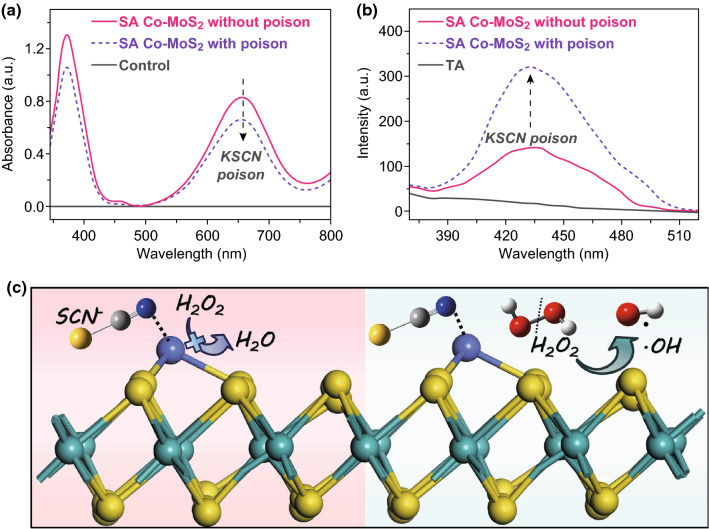
Changes in the **a** UV–Vis spectrum and **b** fluorescence spectrum when SA Co–MoS_2_ was poisoned with 10 mM KSCN. **c** The corresponding illustration of the poison’s influences. Red spheres: O; gray spheres: C; white spheres: H; light-blue spheres: N. (Color figure online)

To better understand the role played by SA Co in determining the catalytic efficiency, we explored the influence of thiocyanate ions (SCN^−^) on the peroxidase-like activity of SA Co–MoS_2_ because SCN^−^ could form a stable chelate complex with metal-centered catalytic sites [49, 50]. Interestingly, an appreciable decrease in the catalytic activity was observed based on the changes in the UV–Vis absorbance at 652 nm (Fig. 3a) and the electrochemical response (Fig. S11). Conversely, a significant enhancement in the fluorescence intensity at 425 nm was observed, as shown in Fig. 3b, indicating that more ·OH was generated in the system. The above phenomena demonstrated that SA Co was one of the major active centers responsible for the peroxidase-like activity. Blocking of SA Co caused more H_2_O_2_ to be decomposed by MoS_2_ than by the poisoned SA Co sites, as illustrated in Fig. 3c. This result further clarified that the introduction of SA Co did not enhance ·OH generation or decompose H_2_O_2_ by the Fenton reaction, and more investigations should be performed. Additionally, no obvious poisoning of the MoS_2_ catalyst by SCN^-^ was observed (Fig. S12) due to the saturated coordination and low stability constant of molybdenum atoms. The other control experiments demonstrated that the peroxidase-like activity of SA Co–MoS_2_ did not result from the effects of dissolved oxygen (Fig. S13a) or leached cobalt ions (Fig. S13b) [48].

### Mechanism of the Peroxidase-like Activity of SA Co–MoS_2_

**Fig. 4:**
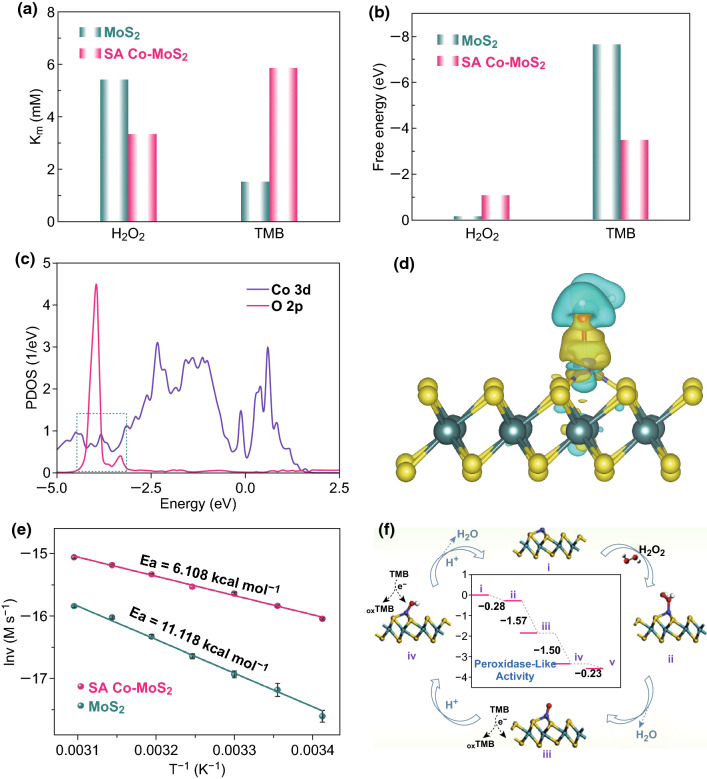
**a** The experimental *K*_m_ values and **b** DFT-calculated adsorption energies of TMB and H_2_O_2_ on MoS_2_ and SA Co–MoS_2_. **c** Theoretical PDOS calculated for H_2_O_2_ on SA Co–MoS_2_. **d** Charge density difference for H_2_O_2_ adsorption on the Co/MoS_2_ monolayer. Regions of electron accumulation and depletion are displayed in blue and yellow, respectively. The isosurface value is ± 0.02 electrons/a.u.^3^. **e** Logarithmic reaction rate over MoS_2_ and SA Co–MoS_2_ as a function of the reciprocal temperature. **f** DFT-calculated reaction energy diagram of H_2_O_2_ dissociation on SA Co–MoS_2_. (Color figure online)

The Michaelis–Menten constant (*K*_m_) was calculated for the MoS_2_ and SA Co–MoS_2_ catalysts toward different substrates (TMB and H_2_O_2_) based on the experimental results of a kinetic analysis (Fig. 4a). *K*_m_ is an indicator of the binding affinity between enzymes and substrates. A lower *K*_m_ value represents a stronger affinity and vice versa [51]. The apparent *K*_m_ value of SA Co–MoS_2_ with H_2_O_2_ was lower than that of MoS_2_ with H_2_O_2_. To explore the internal factors affecting this phenomenon, we also calculated the adsorption energies (*E*_ads_) of H_2_O_2_ and TMB on MoS_2_ and SA Co–MoS_2_ by means of density functional theory (DFT) computations. SA Co–MoS_2_ possessed a higher affinity for H_2_O_2_, while MoS_2_ preferred to adsorb TMB, as shown in Fig. 4b, which is in good agreement with the trend of *K*_m_ values determined by experiments. Moreover, MoS_2_ is negatively charged and prefers interactions with positively charged TMB by electrostatic adsorption [42]. These results also indicated that the H_2_O_2_ molecule could be weakly adsorbed on pristine 2H phase MoS_2_ monolayers, with an *E*_ads_ of -0.10 eV (Fig. 4b). When considering Δ*E*_zpe_ and entropy, the Δ*G* of H_2_O_2_ adsorption on the MoS_2_ monolayers was 0.71 eV, indicating that the H_2_O_2_ molecule would easily escape from the surface. Thus, the MoS_2_ monolayers would exhibit poor catalytic performance in the peroxidase-like reaction. In contrast, the H_2_O_2_ molecule could be chemisorbed on SA Co–MoS_2_ with an *E*_ads_ value of − 1.09 eV and a Δ*G* value of − 0.28 eV, which led to the formation of one Co–O bond with a length of 1.95 Å (Fig. 4b). The strong adsorption of the H_2_O_2_ molecule on SA Co–MoS_2_ could also be verified by the computed partial density of states (PDOSs, Fig. 4c), from which obvious hybridization between Co 3d orbitals and O 2p orbitals can be observed. In addition, approximately 0.32 electrons were transferred from H_2_O_2_ molecules to the Co-anchored MoS_2_ monolayers, mainly accumulating in the Co–O bond. Thus, H_2_O_2_ could be sufficiently activated by SA Co–MoS_2_ (Fig. 4d), facilitating the subsequent peroxidase-like reaction.

The different *E*_ads_ values for MoS_2_ and SA Co–MoS_2_ were attributed to the different catalytic pathways in the peroxidase-like catalytic reaction. (1) Due to the stronger affinity toward H_2_O_2_ and the high redox potential of Co^3+^/Co^2+^ (1.808 V), we proposed that SA Co can serve as an electron transfer mediator. Cytochrome *c* (cyt *c*) is an active electron transporter in biological oxidation, which can be used to verify the accepting electron capability of SA Co–MoS_2_. The two original UV–Vis absorption peaks at 520 and 550 nm attributed to cyt *c* in a reduced state disappeared with the evolution of a new peak at 530 nm for the oxidized cyt *c* after the addition of SA Co–MoS_2_ without or with pure nitrogen for 0.5 h. All these results indicated that SA Co–MoS_2_ was able to obtain electrons from cyt *c* and did not depend on dissolved oxygen (Fig. S14) [44, 46, 52]. (2) The intrinsic enzyme-mimicking property of the MoS_2_ support may result from the occurrence of Fenton-like reactions because the support was more likely to attract TMB (Fig. 4a, b). The above conclusion provided further evidence to support the results of our fluorescence and poisoning experiments and our hypothesis, as shown in Fig. 3. Based on the logarithmic Arrhenius plots (Fig. 4e), the activation energies (*E*_*a*_) were calculated to be 11.12 ± 0.51 (MoS_2_) and 6.11 ± 0.67 kcal mol^−1^ (SA Co–MoS_2_). These experimental results verified that SA Co–MoS_2_ effectively reduced the energy barrier. The free energy profile and the corresponding atomic configurations of various states are presented in Figs. 4f and S15. Our results indicated that the adsorbed H_2_O_2_ molecule dissociated into O^*^ species and one H_2_O molecule [53, 54]. This process was downhill in the free energy profile by 1.57 eV. After the release of the formed H_2_O molecule, the remaining O atom, bound to the Co site with a Co–O length of 1.63 Å, reacted with one proton that originated from the TMB to form an OH species located at the Co site with a Co–O bond of 1.76 Å. Remarkably, this hydrogenation process was exothermic, with a Gibbs energy value (*∆G*) of − 1.50 eV. Finally, the adsorbed OH group reacted with one proton to form a second H_2_O molecule. This final step was also downhill in the free energy profile by 0.23 eV. Overall, each elementary step in the whole peroxidase-like reaction was exothermic in the free energy profile, suggesting the high catalytic activity of a single Co atom supported on the MoS_2_ monolayer. In addition, the MoS_2_ base of SA Co–MoS_2_ was converted from 2H phase to distorted 1T phase, which also contributed to the catalytic efficiency improvement. As shown in Fig. S16a, b, the peroxidase-like activity of MoS_2_ was enhanced through the phase transformation from 2H to 1T phase, which was confirmed by the UV–Vis spectra and electrochemical responses. The strengthened peroxidase-like catalytic activity of 1T-MoS_2_ was attributed to the improvement in conductivity (Fig. S16c) [55], the decrease in the Gibbs energies (Δ*G*), and the negative thermodynamic energy (Δ*U*) compared with the 2H-MoS_2_ (Fig. S17). All of these intrinsic and unique characteristics contributed to the high-efficiency peroxidase-like activity of SA Co–MoS_2_.

### Application of the Peroxidase-like Activity of SA Co–MoS_2_

**Fig. 5:**
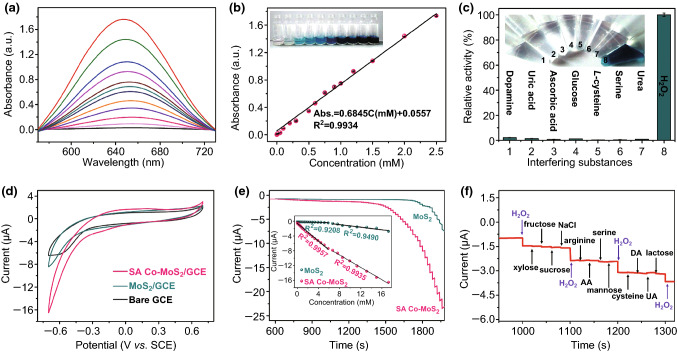
**a** Changes in the UV–Vis absorbance spectra in the presence of different concentrations of H_2_O_2_. **b** The corresponding linear calibration plots. **c** The selectivity of the SA Co–MoS_2_ peroxidase-like catalytic activity. **d** CV responses of SA Co–MoS_2_/GCE, MoS_2_/GCE, and bare GCE in the presence of 0.1 mM H_2_O_2_ in 0.01 M PBS (pH = 7.4). **e** Amperometric response recorded at the MoS_2_/GCE and SA Co–MoS_2_/GCE after the successive addition of H_2_O_2_ to 0.01 M PBS (pH = 7.4) at − 0.50 V. The inset is an enlargement of the low-concentration region (inset: the calibration curve of the MoS_2_/GCE and SA Co–MoS_2_/GCE as a function of the H_2_O_2_ concentration). **f** The selectivity of the SA Co–MoS_2_ composite material

Sensitive and effective biosensors for colorimetric and electrochemical H_2_O_2_ determination were constructed on the basis of SA Co–MoS_2_ peroxidase-like nanozymes [56]. As presented in Fig. 5a, the intensity of the absorption spectra increased with the concentration of H_2_O_2_ with a satisfactory linear relationship ranging from 1 μM to 2.5 mM (Fig. 5b). This established colorimetric method also exhibited specific selectivity, good reproducibility, and high long-term storage stability for H_2_O_2_ (Figs. 5c and S18). In addition, the electrocatalytic activity of SA Co–MoS_2_ toward H_2_O_2_ presented significantly enhanced CV responses (Fig. 5d). Compared with the relevant electrochemical H_2_O_2_ sensors (Table S3), the SA Co–MoS_2_-modified electrode possessed a more extended linear current response ranging from 50 nM to 17.241 mM (Fig. 5e) and a lower detection limit of 10 nM (Fig. S19a) with high selectivity (Fig. 5f), good reproducibility, and stability (Fig. S19b-d). The high stability of SA Co–MoS_2_ was confirmed by X-ray absorption fine structure (XAFS) analysis after the tests (Fig. S20), which indicated that SA Co–MoS_2_ could be used as an excellent enzyme mimic. The underlying source of the remarkable performance could be the balancing of interactions between the catalyst and reaction substrates upon the introduction of SA Co, which adhered to the Sabatier principle of heterogeneous biocatalysis quite well [41, 57].

## Conclusions

In summary, we first demonstrated that SA Co–MoS_2_ is an excellent peroxidase mimic and revealed the occurrence of different mechanisms between the single-atom metal center and support, which provided a complementary strategy for achieving synergistic peroxidase-like properties. This work will not only open the door to another practical frontier of SACs but also offer an important new strategy for designing hybrid nanozymes for biocatalysis.

## Electronic supplementary material

Below is the link to the electronic supplementary material.

Supplementary material 1 (PDF 1923 kb)
