# Supplementary material for: Revealing the Intrinsic Peroxidase-Like Catalytic Mechanism of Heterogeneous Single-Atom Co–MoS2
Source: Nanomicro Lett. 2019 Nov 22;11:102. doi: 10.1007/s40820-019-0324-7 (PMC7770872; doi:10.1007/s40820-019-0324-7)
Supplement: Supplementary file 1 — Supplementary material 1 (PDF 1923 kb) [file 40820_2019_324_MOESM1_ESM.pdf]

Supporting Information for

Revealing the Intrinsic Peroxidase-Like Catalytic Mechanism of Heterogeneous Single-Atom Co-MoS_2

Ying Wang[1, †], Kun Qi[1, 2, †], Shansheng Yu[1], Guangri Jia[1], Zhiliang Cheng[4], Lirong Zheng[5], Qiong Wu[1], Qiaoliang Bao[2], Qingqing Wang[6], Jingxiang Zhao[3, *], Xiaoqiang Cui[1, *], Weitao Zheng[1]

[1]Key Laboratory of Automobile Materials of MOE, School of Materials Science and Engineering, Jilin University, 2699 Qianjin Street, Changchun 130012, People's Republic of China

[2]Department of Materials Science and Engineering, and ARC Centre of Excellence in Future Low-Energy Electronics Technologies (FLEET), Monash University, Clayton, Victoria 3800, Australia

[3]Key Laboratory of Photonic and Electronic Bandgap Materials, Ministry of Education, and College of Chemistry and Chemical Engineering, Harbin Normal University, Harbin 150025, People's Republic of China

[4]Department of Bioengineering, University of Pennsylvania, 210 South 33rd Street, 240 Skirkanich Hall, Philadelphia, PA 19104, USA

[5]Beijing Synchrotron Radiation Facility, Institute of High Energy Physics, Chinese Academy of Sciences, Beijing 100190, People's Republic of China

[6]School of Chemistry and Chemical Engineering, MOE Key Laboratory of Micro-System and Micro-Structure Manufacturing, Harbin Institute of Technology, Harbin 150001, People's Republic of China

[†]Ying Wang and Kun Qi contributed equally to this work

*Corresponding authors. E-mail: xqcui@jlu.edu.cn (Xiaoqiang Cui); xjz_hmily@163.com (Jingxiang Zhao)

Supplementary Figures and Tables

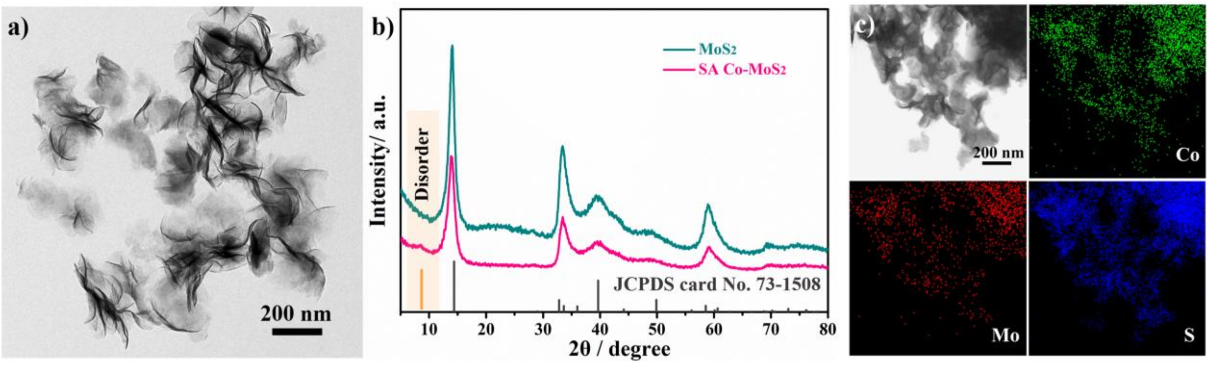

Fig. S1 (a) TEM image of SA Co-MoS_2. No Co nanoparticles or clusters were observed. **(b)** XRD pattern of MoS_2 and SA Co-MoS_2. **(c)** The elemental mapping images of SA Co-MoS_2

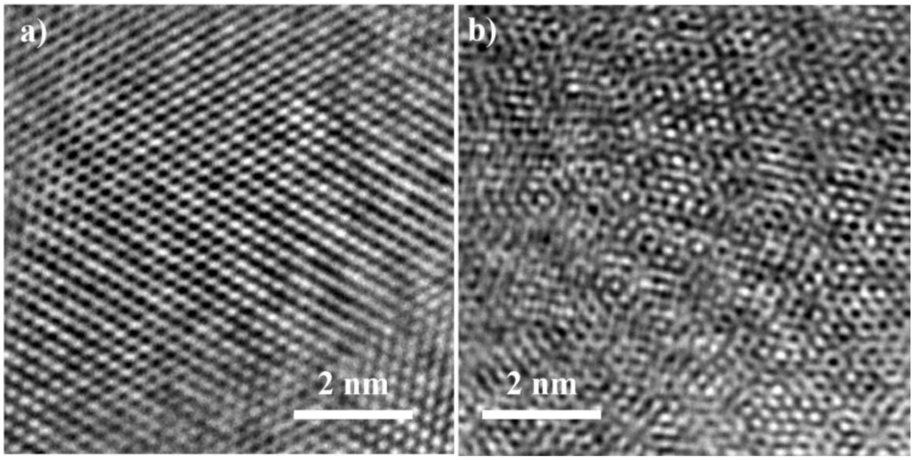

Fig. S2 HRTEM images of **(a)** MoS_2 and **(b)** SA Co-MoS_2

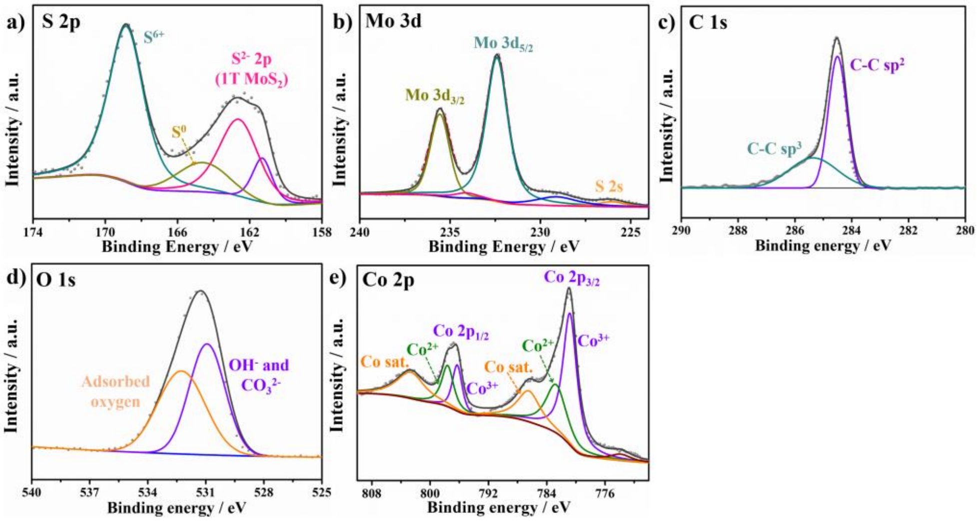

Fig. S3 High-resolution **(a)** S 2p, **(b)** Mo 3d, **(c)** C 1s, **(d)** O 1s, and **(e)** Co 2p XPS spectra of SA Co-MoS_2

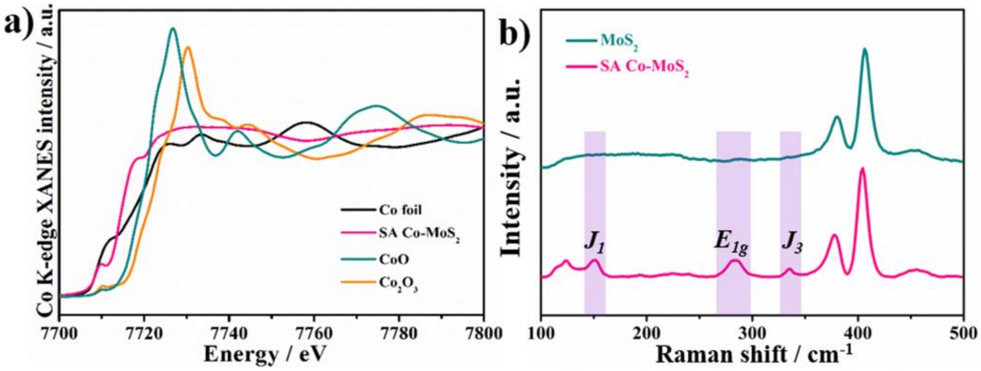

Fig. S4 **(a)** Normalized Co K-edge XANES spectra of Co foil, CoO, Co_2O_3, and SA Co-MoS_2. **(b)** Raman spectra of MoS_2 and SA Co-MoS_2

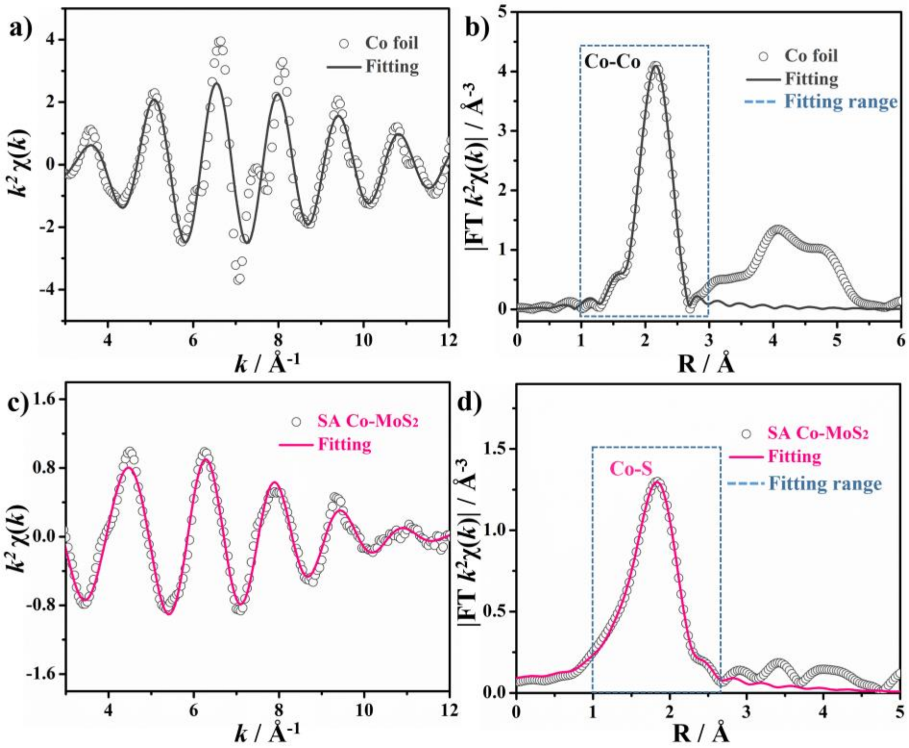

Fig. S5 (a) *k* space fitting curve and **(b)** FT-EXAFS fitting curves of the Co foil. **(c)** *k* space fitting curve and **(d)** FT-EXAFS fitting curves of the SA Co-MoS_2

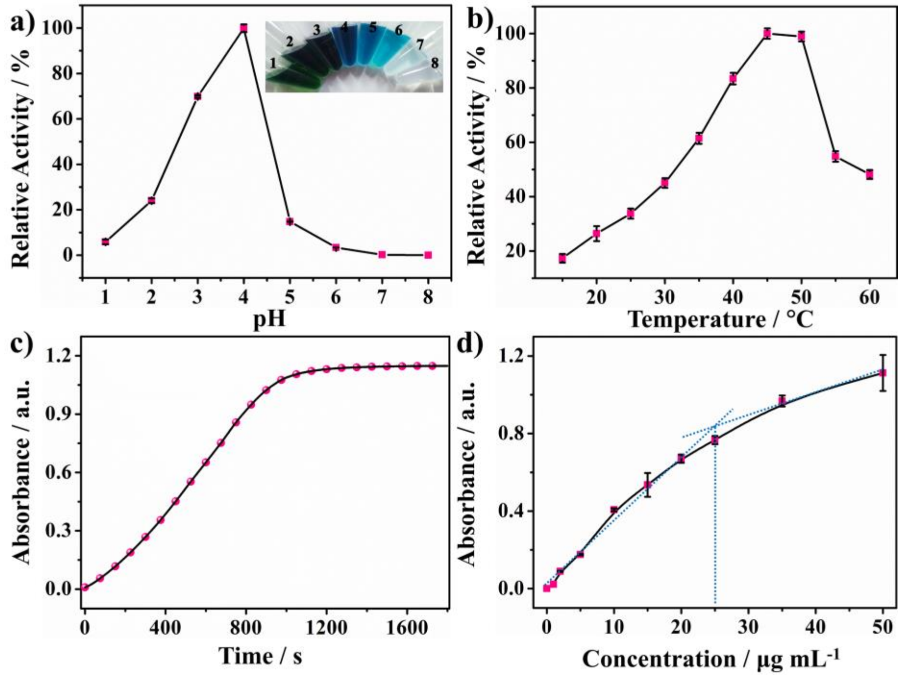

Fig. S6 Effects of different **(a)** pH values of the acetate buffer (inset: Photograph of the corresponding samples), **(b)** incubation temperatures, **(c)** incubation times, and **(d)** concentrations of SA Co-MoS_2

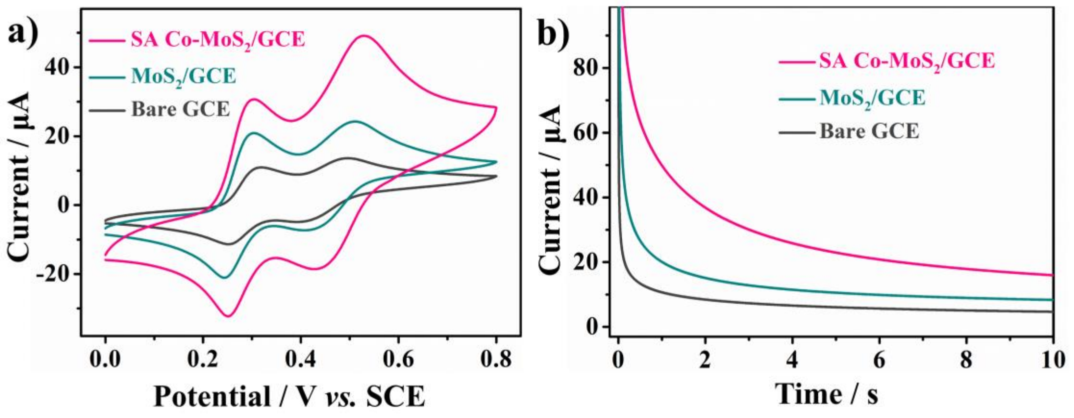

Fig. S7 (a) CV curves and **(b)** chronoamperometric curves of 0.1 M pH 4.0 HAc-NaAc buffer containing 5.0 mM TMB and 1.0 mM H_2O_2 measured by a GCE, MoS_2/GCE and SA Co-MoS_2/GCE

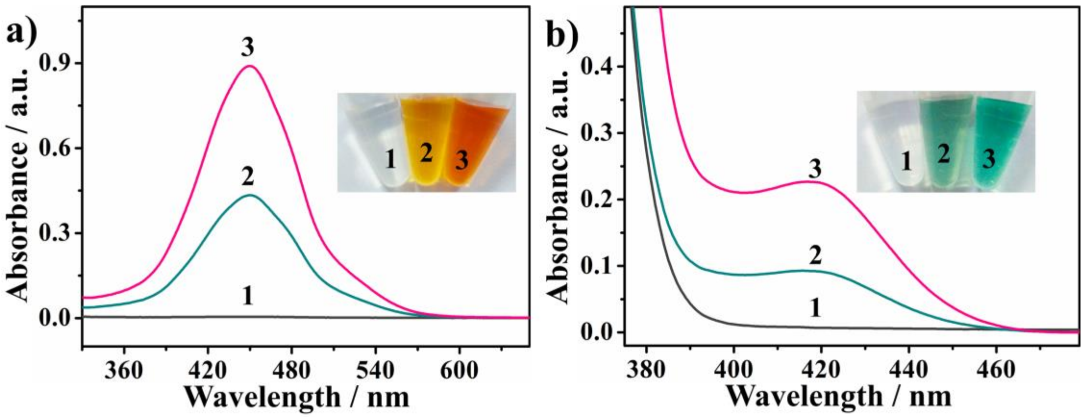

Fig. S8 The typical UV-Vis spectra in different reaction systems with OPD **(a)** and ABTS **(b)** as peroxidase substrates: (1) substrate + H_2O_2, (2) substrate + H_2O_2 + MoS_2, and (3) substrate + H_2O_2 + SA Co-MoS_2 (inset: optical image showing the corresponding colour changes)

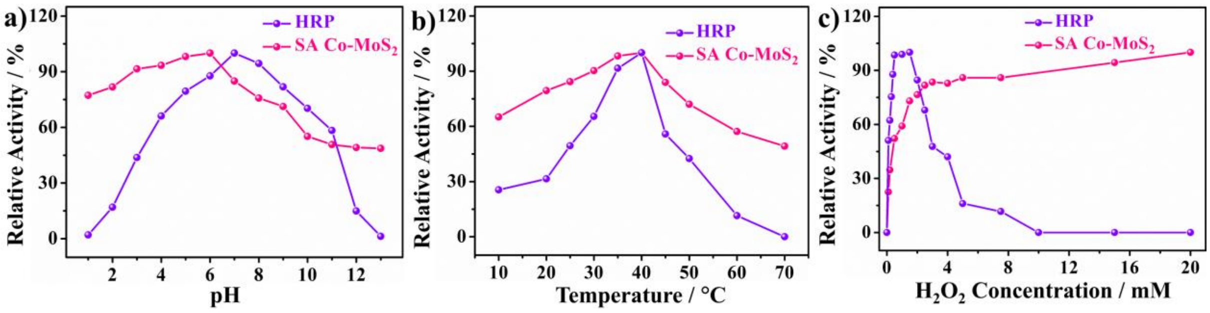

Fig. S9 The comparison of the **(a)** pH, **(b)** temperature, and **(c)** H_2O_2 tolerances of SA Co-MoS_2 and HRP

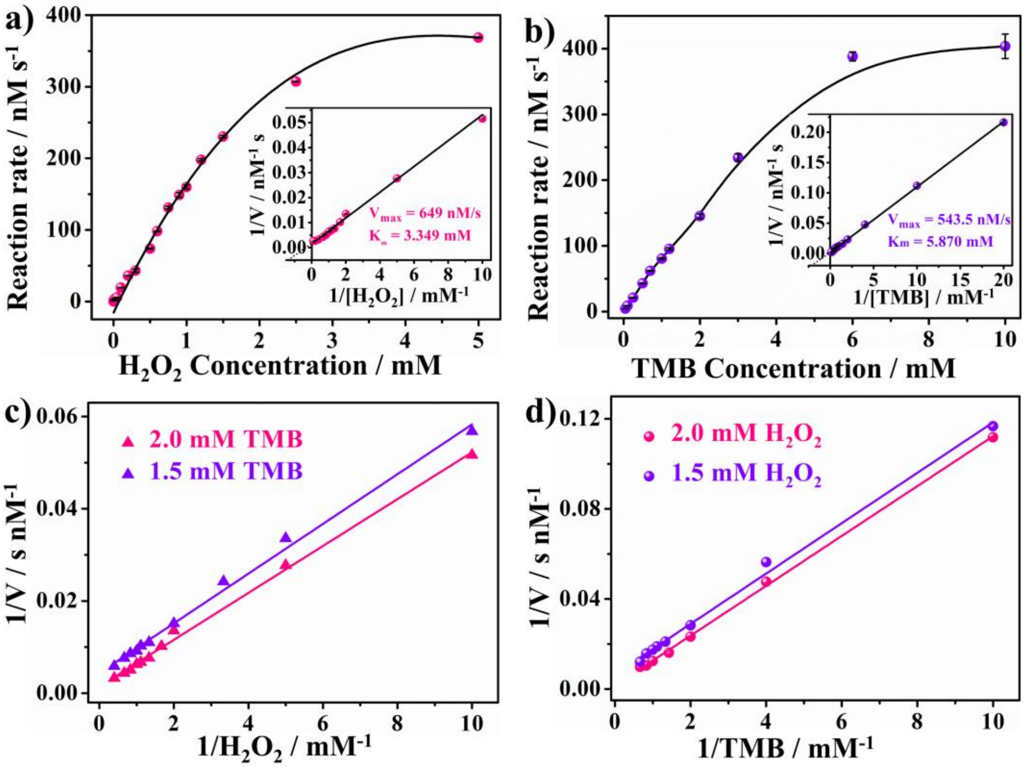

Fig. S10 Kinetic analysis of the reaction of SA Co-MoS_2 with H_2O_2 **(a)** or TMB **(b)**. The insets in panels **(a)** and **(b)** show the corresponding double-reciprocal plots for calculation of the enzyme kinetic parameters by the Michaelis-Menten equation. Double-reciprocal plots of the SA Co-MoS_2 activity with the concentration of one substrate (H_2O_2 **(c)** or TMB **(d)**) fixed and the other varied

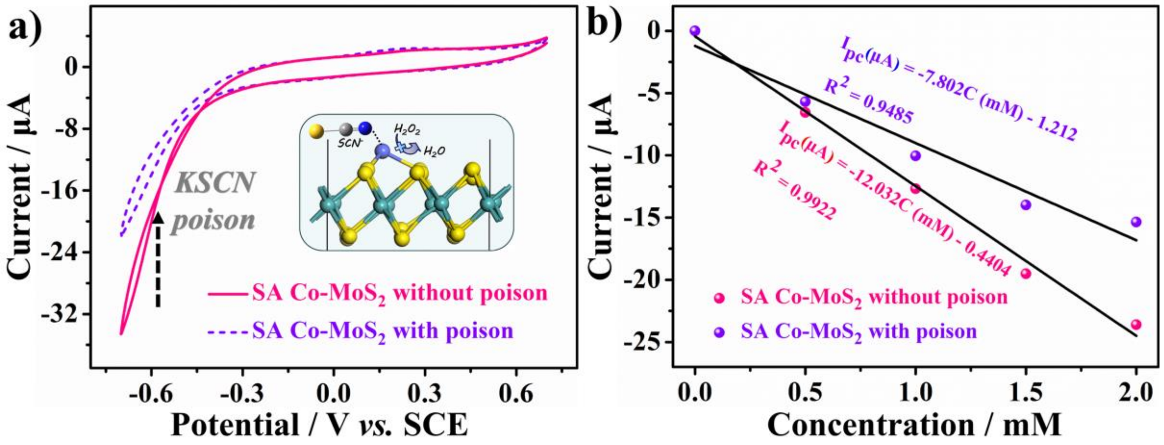

Fig. S11 (a) The CV responses of the SA Co-MoS_2/GCE in N_2-saturated 0.01 M PBS (pH = 7.4) containing 3.0 mM H_2O_2 without and with 10 mM KSCN poison. The inset is an illustration showing the blocking of the cobalt centre by the SCN^- ion. **(b)** The calibration curves for H_2O_2 concentrations from 0.00 to 2.0 mM at −0.7 V

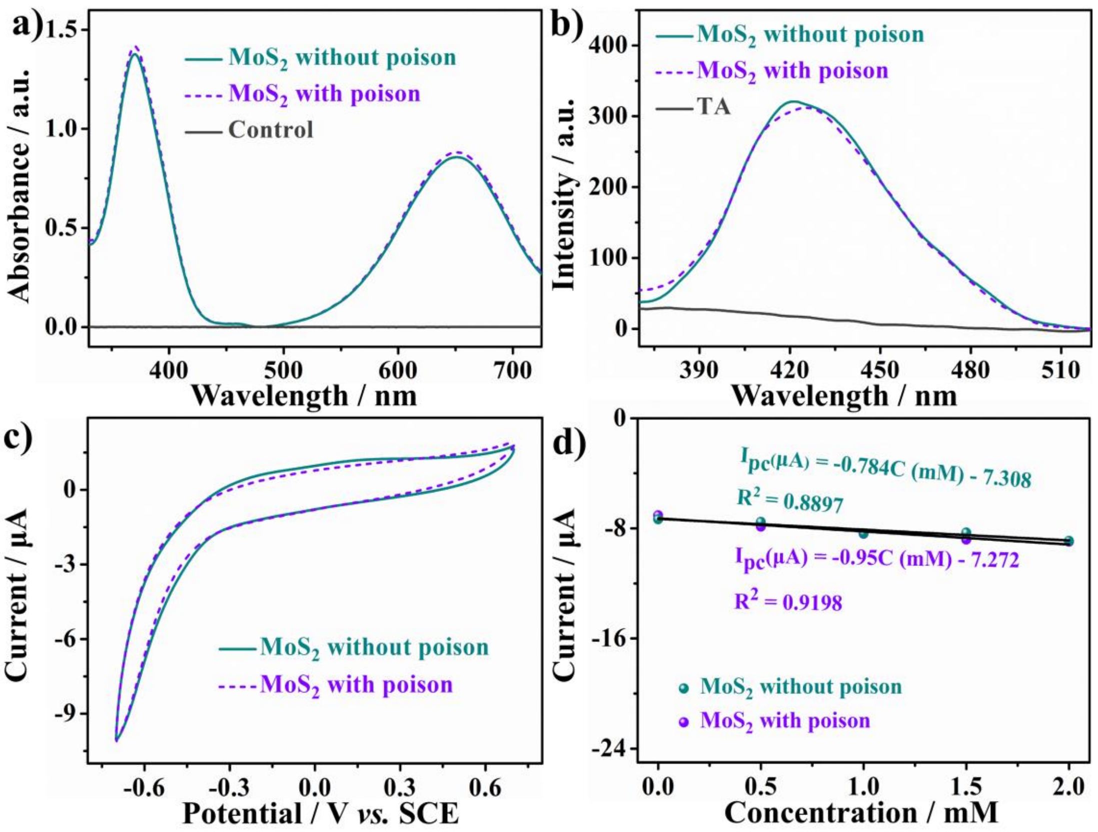

Fig. S12 Changes in the UV-Vis absorbance **(a)** and fluorescence spectra **(b)** when the MoS_2 catalyst was poisoned with 10 mM KSCN. **(c)** The CV response of the MoS_2/GCE in N_2-saturated 0.01 M PBS (pH = 7.4) containing 3.0 mM H_2O_2 without and with 10 mM KSCN poison. **(d)** The calibration curves for H_2O_2 concentrations from 0.00 to 2.0 mM at −0.7 V

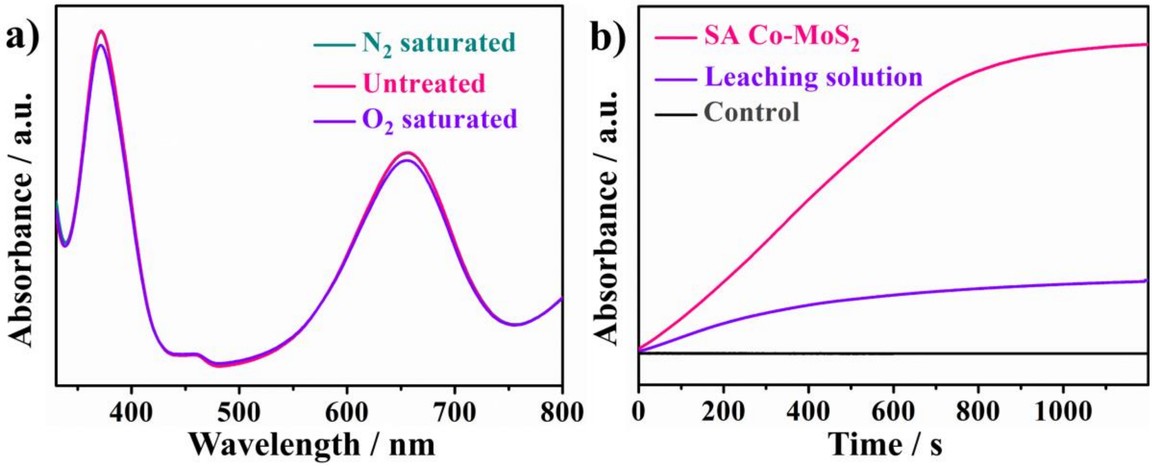

Fig. S13 (a) Effect of dissolved oxygen. **(b)** Demonstration that the peroxidase-like activity of SA Co-MoS_2 does not result from cobalt ion leaching

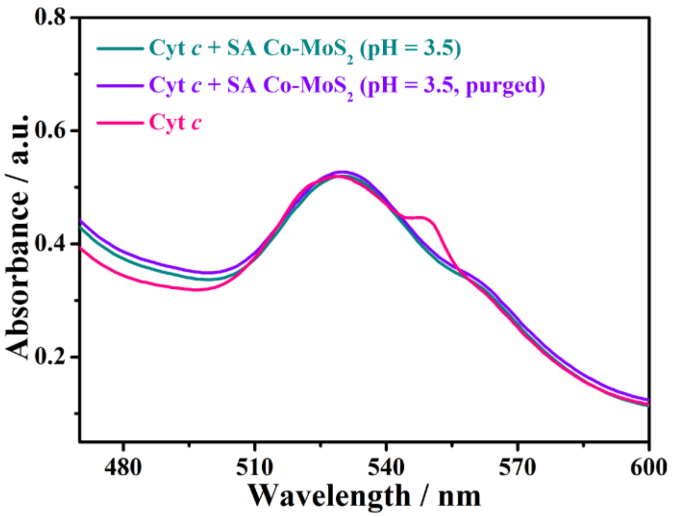

Fig. S14 UV−Vis spectrum of origin Cyt *c*, Cyt *c* reacted with SA Co-MoS$_2$ and Cyt *c* reacted with SA Co-MoS$_2$ under deoxygenated condition

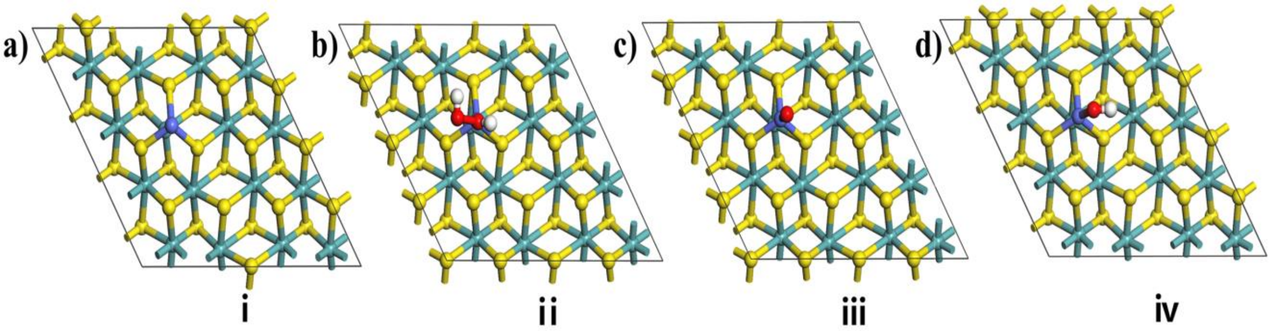

Fig. S15 The top views of H_2O_2 decomposition on the surface of distorted 1T MoS$_2$ slab with a single Co atom absorbed

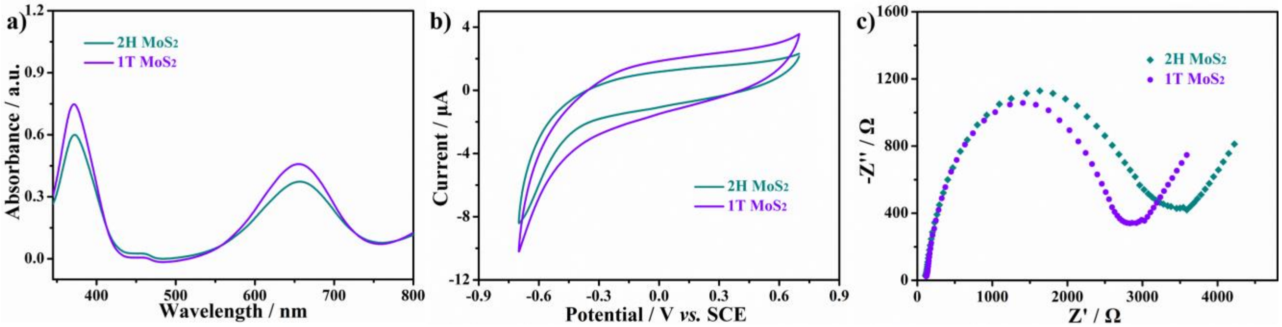

Fig. S16 The comparation between the 2H MoS$_2$ and 1T MoS$_2$ for changes in **(a)** UV-Vis absorbance, **(b)** CV response in N_2-saturated 0.01 M PBS (pH = 7.4) containing 1.0 mM H_2O_2, and **(c)** the typical Nyquist plots in 5 mM $[Fe(CN)_6]^{3-/4-}$ and 0.1 M KCl with frequency varied from 100 kHz to 0.1 Hz at open circuit potential

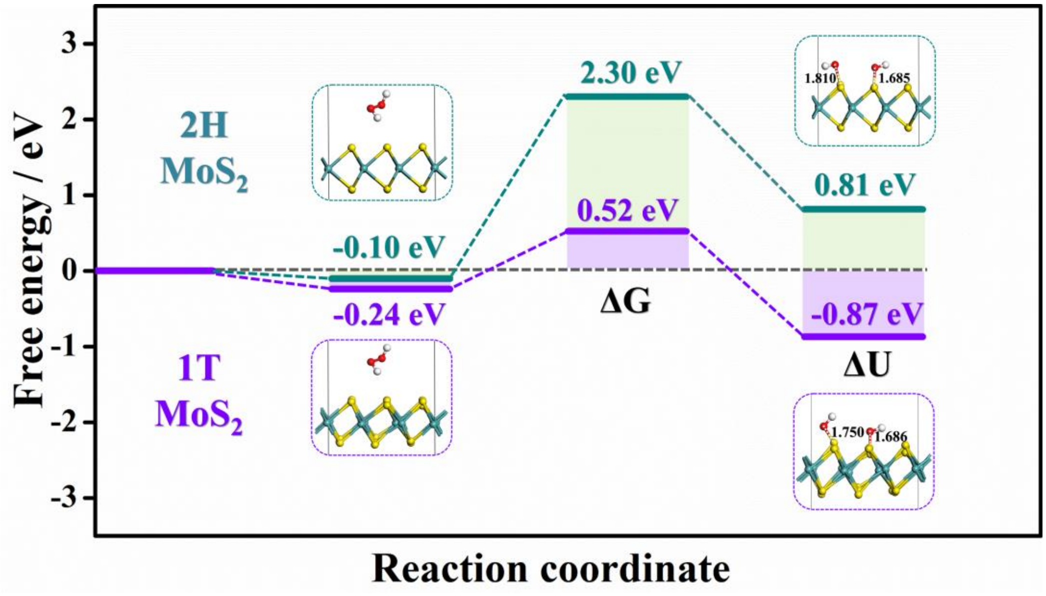

Fig. S17 DFT-calculated reaction energy diagram of H_2O_2 dissociation on 2H MoS_2 and 1T MoS_2

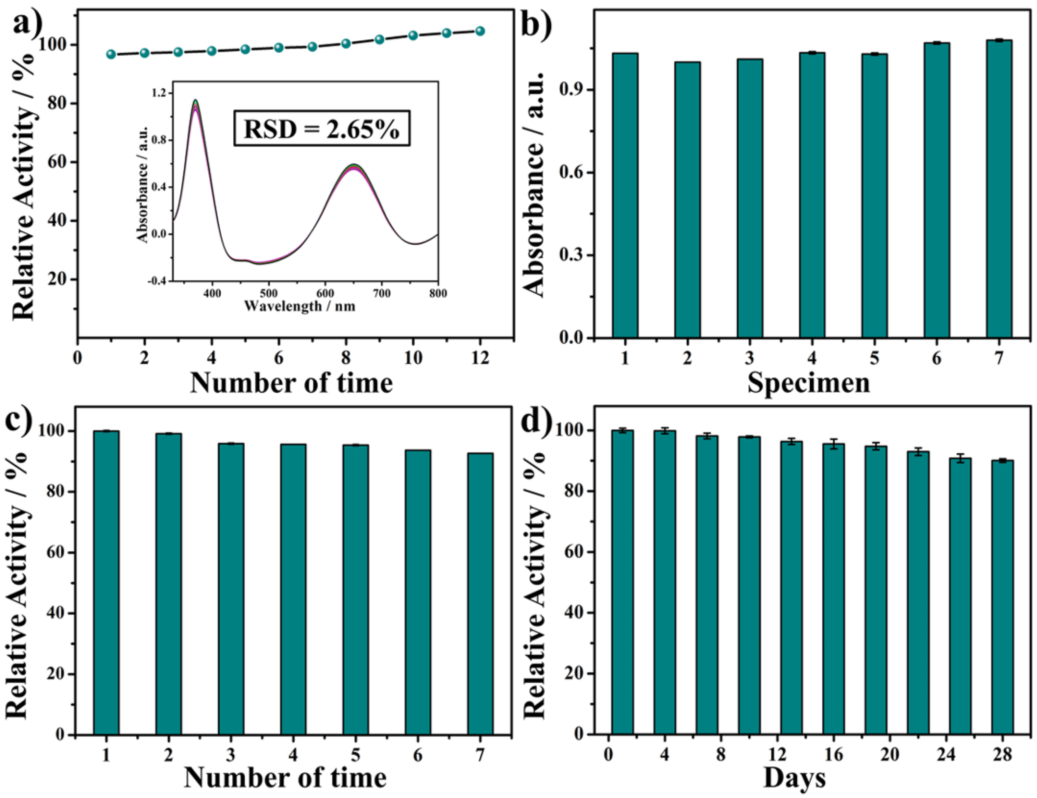

Fig. S18 The reproducibility of the method **(a)**, samples **(b)**, and catalyst **(c)**. Long-term storage stability **(d)** of the SA Co-MoS_2 catalytic activity

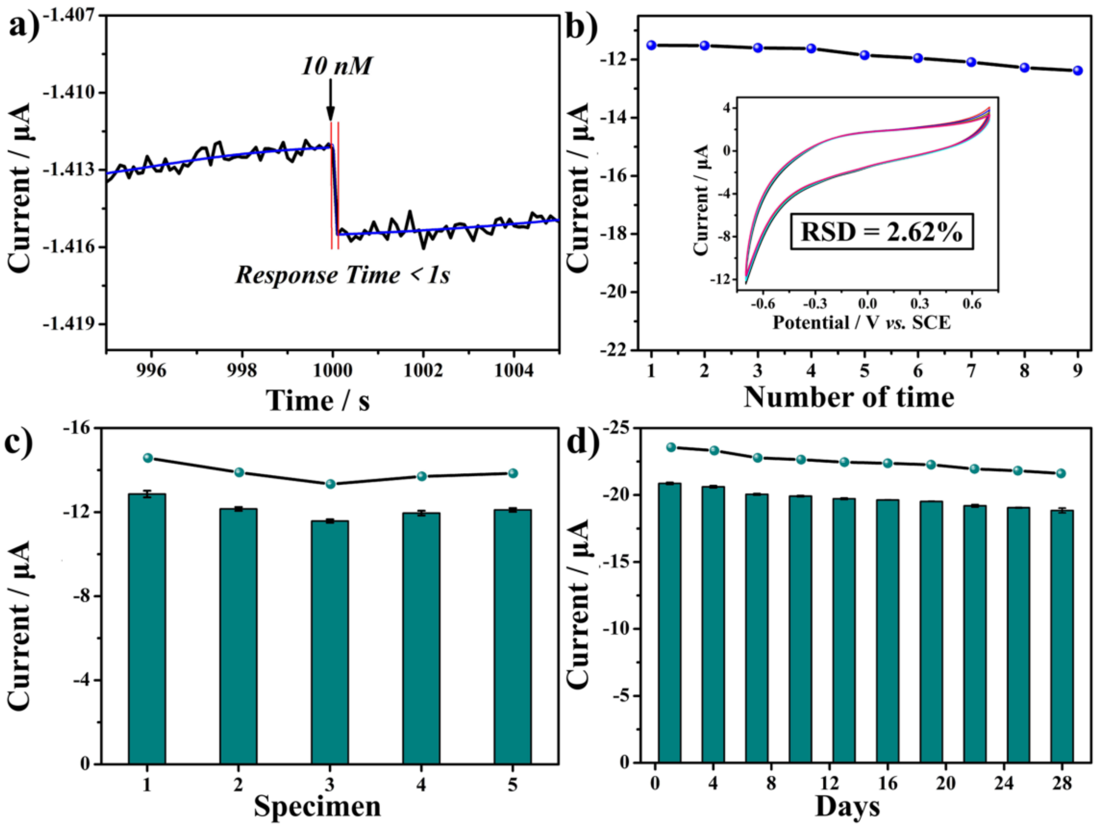

Fig. S19 **(a)** The detection limit (S/N = 3) of the SA Co-MoS$_2$ composite material. The electrode reproducibility of **(b)** 9 consecutive current measurements by the same electrode and **(c)** the electrode-to-electrode reproducibility for five separate electrodes in 0.5 mM H_2O_2. **(d)** The long-term stability of the sensor at a H_2O_2 concentration of 1.0 mM

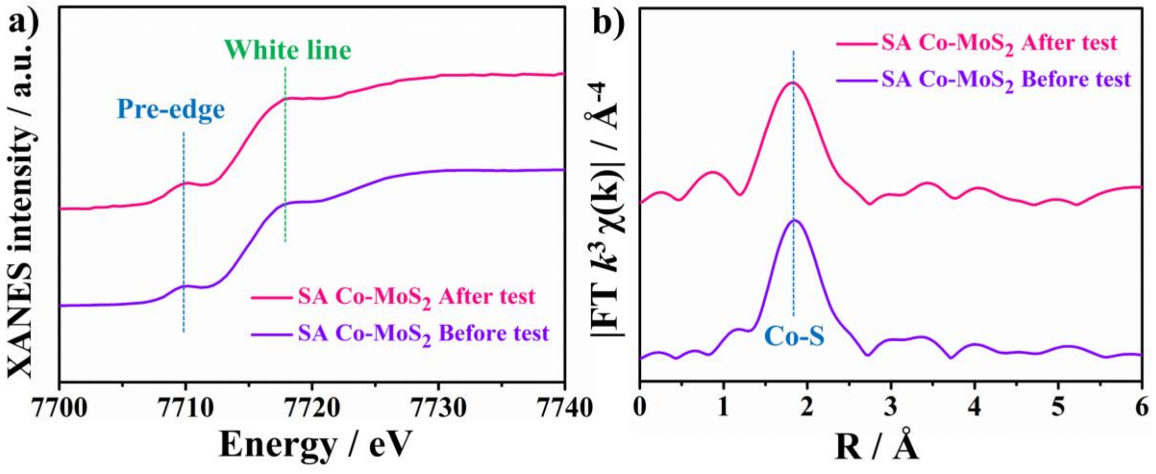

Fig. S20 SA Co-MoS$_2$ before and after the peroxidase-like catalytic reaction. **(a)** Co K-edge XANES spectra and **(b)** FT-EXAFS spectra

Table S1 Mo *K*-edge EXAFS curves fitting parameters

Sample	Path	Coordination Number	Bond length R (Å)	Bond disorder σ^2 ($\times 10^{-3}$Å^2)	ΔE_0 (eV)	R (%)
**Co foil**	Co-Co	12*	2.50±0.01	6.4±0.3	7.8±0.4	0.001
**SA Co-MoS$_2$**	Co-S	3.4±0.6	2.23±0.02	7.6±2.2	−1.5±1.6	0.001

Table S2 Comparison of the apparent kinetic parameters of SA Co-MoS$_2$, MoS$_2$, and HRP

Catalyst	Substance	K_m (mM)	V_{max} (M s^{-1})	References
**SA Co-MoS$_2$**	TMB	5.870	5.44×10^{-7}	This work
	H_2O_2	3.349	6.49×10^{-7}	
**MoS$_2$**	TMB	1.531	4.61×10^{-8}	This work
	H_2O_2	5.430	2.70×10^{-7}	
**HRP**	TMB	0.434	10.0×10^{-8}	Nat. Nanotechnol. 2007, 2, 577
	H_2O_2	3.702	8.71×10^{-8}	

Table S3 Comparison of different molybdenum disulphide-based electrochemical sensors for the determination of H_2O_2

Sensing platform	Linear range	LOD (nM)	References
SA Co-MoS$_2$	50 nM − 5.845 mM<br>5.845 mM − 17.241 mM	10	This work
Pt/MoS$_2$/Ti	10 µM – 160 µM	870	J. Electroanal. Chem. 2018, 15, 274
MoS$_2$-ICPC	20 µM – 300 µM	11800	J. Electroanal. Chem. 2018, 15, 429
MoS$_2$/CC	5 µM – 235 µM<br>435 µM – 3000 µM	1000	Chem. Commun., 2019, 55, 9653
Pt-Pd/MoS$_2$	10 µM – 80 µM	3400	Microchim. Acta, 2018, 185, 399
interlayer-expanded MoS$_2$	0.23 µM – 2200 µM<br>2200 µM – 14220 µM	200	Nanoscale 2019, 11, 6644
MoS$_2$-GSSG NSs	0 µM − 50 µM	510	Chem. Eur. J. 2018, 24, 15868
PtW-MoS$_2$	1 µM – 200 µM	1710	Biosens. Bioelectron, 2016, 80, 601
